# Immune Response Associated Hepatotoxicity in Hemophilia Gene Therapy: Mechanisms, Management, and Challenges

**DOI:** 10.14740/jh2161

**Published:** 2026-02-20

**Authors:** Chang Cheng Zheng

**Affiliations:** Department of Hematology, Anhui Provincial Hospital, Hefei 230001, China. Email: zhengchch1123@ustc.edu.cn

**Keywords:** AAV gene therapy, Hemophilia, Hepatotoxicity, Immune response activation, Corticosteroids

## Abstract

Adeno-associated virus (AAV)-based gene therapy offers the potential for long-term functional cure in patients with hemophilia A and B. However, immune responses triggered by the vector capsid or transgene product, leading to hepatotoxicity, represent a major challenge to the long-term stability of transgene expression and treatment safety. Based on reported clinical trials of hemophilia gene therapy, this review delves into the mechanisms of immune response activation following AAV gene therapy and summarizes the clinical features and monitoring strategies for hepatotoxicity, which primarily manifests as asymptomatic transaminase elevation. It highlights the roles of patient selection, vector optimization, and current clinical management strategies centered on corticosteroids in preventing and managing immune responses to stabilize transgene expression and prevent the decline of clotting factor levels. Furthermore, the review discusses potential differences between hemophilia A and B gene therapy, challenges such as long-term safety concerns (including tumorigenicity risk) and pre-existing immunity, and provides an outlook on future directions including vector engineering, immune modulation, and personalized treatment approaches. The aim is to offer practical insights for clinicians and promote the safer application of hemophilia gene therapy.

## Introduction

Hemophilia, an X-linked inherited bleeding disorder caused by deficiencies in coagulation factor VIII (FVIII, hemophilia A) or IX (FIX, hemophilia B), has traditionally been managed with a treatment paradigm reliant on frequent intravenous infusions of clotting factor concentrates. This strategy has significantly improved patients’ quality of life and life expectancy; however, the burden of lifelong treatment, the risk of breakthrough bleeding, and the potential for inhibitor development (particularly in hemophilia A) remain significant limitations of current factor replacement therapy [1, 2].

*In vivo* gene therapy based on adeno-associated virus (AAV) vectors, with its ultimate goal of achieving sustained, stable endogenous coagulation factor expression following a single treatment, represents a paradigm shift in the management of hemophilia [3, 4]. In recent years, landmark clinical trials have successfully demonstrated that AAV-mediated gene therapy can elevate factor levels in patients with moderate-to-severe hemophilia to the mild or even normal range, thereby significantly reducing or eliminating the need for replacement therapy [5]. However, host immune responses triggered by the AAV vector and the associated immune-mediated hepatotoxicity in the primary target organ, the liver, pose critical challenges to the durability of efficacy and safety of this approach [6]. This hepatotoxicity often manifests clinically as asymptomatic transaminase elevation, but its fundamental consequence is the loss of transgene expression, which can significantly undermine therapeutic success. The underlying mechanism involves a complex cascade, including initial activation of the innate immune system, cross-priming and expansion of AAV capsid-specific and transgene product-specific T cells, and ultimately, the clearance of transduced hepatocytes by cytotoxic T lymphocytes [7–9]. Therefore, a deep understanding of the triggers, core effector mechanisms of this immune response, and its intrinsic link to hepatotoxicity and factor loss, coupled with the establishment of effective strategies for its prevention, monitoring, and management, is essential for the successful transition of hemophilia gene therapy from clinical trials to widespread clinical application.

This review aims to provide a comprehensive reference for the international community of hemophilia gene therapy practitioners. To ensure the comprehensiveness and representativeness of this review, the literature was identified and selected through the following strategy: Combined searches were conducted in the PubMed and Web of Science databases using keywords such as “hemophilia,” “gene therapy,” “immune-mediated liver injury,” “immune-mediated hepatotoxicity,” “autoimmune liver disease,” “liver inflammation,” “AAV vectors,” and their related terms. The search timeframe primarily focused on articles published between January 2011 and October 2025. During the selection process, priority was given to high-quality original research articles, authoritative reviews, and significant clinical trial reports, with attention paid to diverse perspectives and the latest breakthroughs.

## Triggers of the Immune Response

The long-term efficacy and safety of hemophilia gene therapy are significantly constrained by host immune responses triggered by the AAV vector [10, 11]. These responses primarily target the liver, with their core consequences being immune-mediated hepatocyte injury (manifesting as transaminase elevation) and loss of transgene expression. The underlying mechanism involves a cascade encompassing both innate and adaptive immunity.

### AAV capsid protein

The AAV capsid is the primary target for immune recognition. Following proteasomal degradation within transduced hepatocytes, capsid-derived peptides are presented via major histocompatibility complex (MHC) class I molecules, potentially activating CD8^+^ T cells [12, 13]. Evidence indicates that this capsid-specific T-cell response is a major driver of hepatotoxicity. In Nathwani’s study on AAV8-hFIX, patients in the high-dose cohort (2 × 10^12^ vg/kg) exhibited expansion of AAV8 capsid-specific T cells in peripheral blood, coinciding with alanine aminotransferase (ALT) elevation and declining FIX levels [14, 15]. Interferon-γ (IFN-γ) enzyme-linked immunospot (ELISpot) analysis confirmed that these T-cell responses were polyclonal [16]. Similarly, possible associations between capsid-specific T-cell responses and elevated liver enzymes accompanied by declining factor levels were observed in other clinical trials [17–20].

### Vector dose and genome design

The incidence of hepatotoxicity correlates positively with the vector dose [21–24]. It has been postulated that a higher vector dose might enhance transgenic protein expression within hepatocytes [18, 25]; this could raise the antigenic burden presented by these cells, making them more susceptible to targeting and potentially priming or intensifying a T-cell response directed against the transgene product. However, this causality has not been directly established in humans. Furthermore, vector genome design is a critical influencing factor. CpG motifs within the vector genome are potent activators of innate immunity [26–30]. The BAX 335 study (hemophilia B) was the first clinical trial to suggest that high-density CpG motifs, inadvertently introduced during codon optimization, might stimulate innate immunity via Toll-like receptor 9 (TLR9) pathway activation, thereby potentiating the adaptive immune response against the capsid and leading to FIX expression loss [19]. Although subsequent studies, such as TAK-754 (hemophilia A), substantially reduced CpG content (> 90%), all participants still experienced ALT elevation and FVIII expression loss, indicating that cellular immune responses remain difficult to fully avert even with optimized transgene design [31].

### Pre-existing immunity

The prevalence of pre-existing neutralizing antibodies (NAbs) and memory T cells against AAV in the general population are significant considerations [32–37]. Pre-existing NAbs can directly impact initial transduction efficiency, while memory T cells can accelerate immune clearance. Long-term follow-up studies by George et al demonstrated that high-titer NAbs generated after AAV2 infusion can persist for up to 15 years and exhibit broad serotype cross-reactivity, constituting a major barrier to re-administration [38]. Although some serotypes like AAV5 demonstrate better immune evasion (e.g., effective transduction was achieved in the etranacogene dezaparvovec trial with pre-existing AAV5 NAb titers ≤ 1:678) [39], pre-existing immunity remains a crucial factor for patient selection and individualized risk assessment, primarily regarding the risk of suboptimal transduction, therapeutic non-response, or loss of durability, rather than conventional safety risks.

### Immunogenicity of the transgene product and hepatocyte stress

Although FVIII and FIX are self-proteins, their variants (e.g., FIX Padua) or overexpression might be perceived as “non-self” by the immune system. In hemophilia A, the large size and complex structure of FVIII render its overexpression more prone to inducing endoplasmic reticulum (ER) stress and the unfolded protein response (UPR), which could exacerbate hepatocyte damage and immune recognition [40–42]. In animal models, FVIII expression driven by strong promoters can induce UPR, leading to hepatocyte apoptosis [43, 44]. In clinical studies of hemophilia B gene therapy [19, 45], the timing of ALT elevation in certain patients is associated with the post-peak decline of FIX expression, which may involve potential metabolic stress alongside vector-induced immune responses. In studies of valoctocogene roxaparvovec (AAV5-hFVIII-SQ) for hemophilia A [25], typical AAV5 capsid-specific T-cell responses (evidenced by IFN-γ ELISpot signals) and ALT elevations were observed, with 85.8% of participants in the phase 3 GENEr8-1 trial experiencing ALT increases managed by glucocorticoids; however, UPR was not directly confirmed as the primary driver of clinical hepatotoxicity.

## Core Effector Mechanisms of the Immune Response

**Figure 1:**
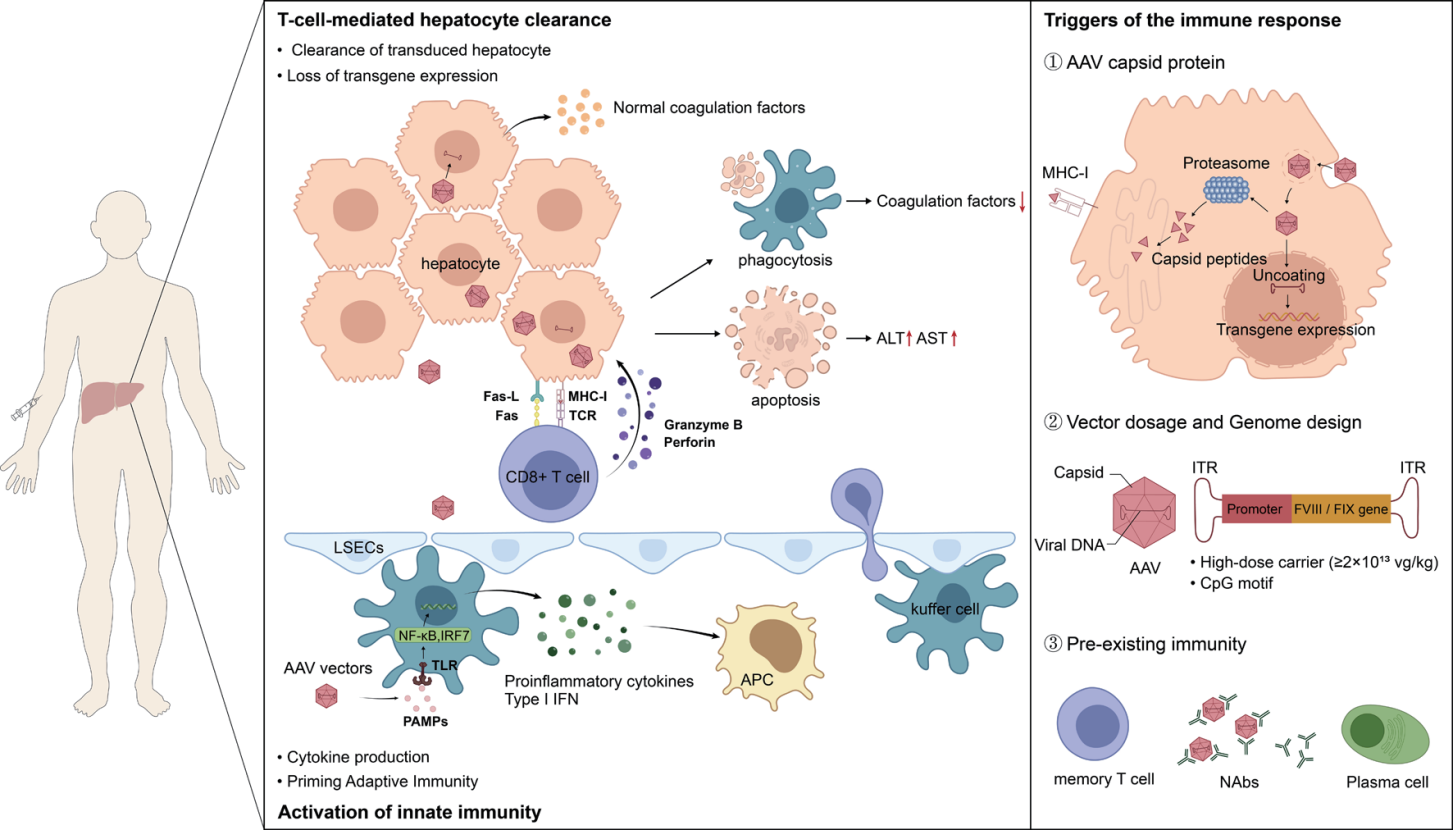
AAV vectors-triggered immunological liver injury and transgene loss. Hepatocyte injury mediated by CD8^+^ cytotoxic T lymphocytes (CTLs) constitutes the central mechanism underlying hepatotoxicity and the loss of coagulation factor expression. Activated AAV capsid-specific CTLs migrate to the liver, where they recognize and attack transduced hepatocytes presenting the corresponding peptide–MHC-I complexes. The attack mechanisms include the release of perforin and granzymes to directly induce hepatocyte apoptosis, as well as Fas/FasL pathway-mediated cell death. This specific killing leads to the elimination of hepatocytes containing the transgene genome, thereby resulting in elevated liver transaminases and decreased coagulation factor expression levels. Furthermore, the innate immune response is the earliest defensive reaction triggered upon exposure to AAV vectors. When AAV vectors are administered intravenously, pathogen-associated molecular patterns (PAMPs) such as viral capsid proteins and unmethylated CpG oligodeoxynucleotides within the vector genome are recognized by Toll-like receptors on the surface of hepatocytes, liver sinusoidal endothelial cells, and resident immune cells in the liver, such as Kupffer cells. This recognition activates downstream signaling pathways, leading to the activation of nuclear factor kappa B (NF-κB) and interferon regulatory factors (IRFs), which in turn promotes the release of type I interferons (IFN-α/β) and various inflammatory cytokines, such as IL-6 and TNF-α. Additionally, AAV capsid proteins, vector dosage and genome design, and pre-existing immunity are all important triggers of immune responses.

The mechanisms underlying hepatotoxicity and the eventual loss of transgene expression following AAV-mediated gene therapy are an area of active investigation. A predominant hypothesis posits that CD8^+^ T cell immunity directed against the AAV capsid is a key driver, leading to the immune-mediated clearance of transduced hepatocytes [13]. This process initiates with innate immune activation following high-dose vector infusion, leading to cross-priming and activation of capsid-specific CD8^+^ T cells, which ultimately mediate the targeted clearance of transduced hepatocytes [46–50]. Vector design (e.g., CpG content), transgene product characteristics (FVIII vs. FIX), and host pre-existing immunity collectively modulate the intensity and manifestation of this process (Fig. 1). However, the potential mechanisms of gene therapy-induced immune-mediated hepatotoxicity discussed in this article synthesize evidence from different levels; it is important to clarify that while some mechanisms have been more deeply elucidated in preclinical models (such as *in vitro* cell experiments or animal models), others have obtained supportive data in human patients or are under validation.

### Innate immune activation and inflammatory milieu establishment

Following AAV vector infusion, pathogen-associated molecular patterns (PAMPs) within the capsid and genome are recognized by TLRs (e.g., TLR2, TLR9) on hepatic immune cells (e.g., Kupffer cells), activating nuclear factor kappa B (NF-κB) and interferon regulatory factor 7 (IRF7) signaling pathways [51, 52]. This leads to the release of type I interferons and pro-inflammatory cytokines (e.g., interleukin-6 (IL-6), tumor necrosis factor-α (TNF-α)) [19]. This innate immune response creates a pro-inflammatory environment that may not only directly suppress transgene expression but, more importantly, significantly enhances the maturation and function of antigen-presenting cells, paving the way for initiating adaptive immunity.

### T cell-mediated clearance of hepatocytes: the direct cause of hepatotoxicity and factor loss

Activated capsid-specific CD8^+^ T cells migrate to the liver and, upon recognizing capsid peptide-MHC-I complexes presented on the surface of transduced hepatocytes, mediate specific killing of these cells [49, 53, 54]. The attack mechanisms involve perforin/granzyme release and induction of apoptosis via the Fas/FasL pathway [55, 56]. This directly results in hepatocyte necrosis (manifesting as elevated ALT/aspartate aminotransferase (AST)) and the elimination of hepatocytes containing the transgene genome (leading to decreased coagulation factor expression levels) [15, 57]. This response typically peaks between 4 and 12 weeks post-infusion, aligning with the clinically observed window of liver enzyme elevation. The effectiveness of corticosteroid therapy in controlling ALT elevation and partially restoring factor levels in most patients strongly supports its immune-mediated nature [14, 58, 59].

### Potential impact of humoral immune responses

High-titer anti-AAV NAbs generated after gene therapy, while having limited impact on the efficacy of the current treatment, severely compromise the possibility of future AAV-based gene therapy [38, 60]. Furthermore, the development of FVIII inhibitors is a major concern in hemophilia A gene therapy. Theoretically, continuously expressed FVIII could break immune tolerance. In the GENEr8-1 trial, transient anti-FVIII antibodies developed in 9.0% of participants, but these did not progress to clinically significant inhibitors [61]. To date, large-scale clinical studies have not reported inhibitor formation, but long-term risk should be monitored.

### Mechanism differences between hemophilia A and B

While the fundamental immune mechanisms underlying hepatotoxicity are similar, notable differences exist. FVIII’s large size and complex biosynthesis make it more prone to inducing ER stress, and its delayed expression kinetics (peak at 20–28 weeks) might prolong the window of immune exposure [20, 47]. FIX is smaller and its expression is relatively rapid and stable. In hemophilia B trials (e.g., BAX 335, FLT180a), immune responses were significantly linked to CpG-driven innate immunity or typical capsid T-cell reactions [17, 19]. In contrast, in hemophilia A trials (e.g., SPK-8011, GENEr8-1), capsid-specific T-cell responses appear more stereotypical, and hepatotoxicity seems more frequent, potentially related to higher vector doses and the inherent expression burden of FVIII [18, 25]. The observed higher risk of hepatotoxicity in hemophilia A is a manifestation of the complex interplay between the intrinsic properties of the FVIII protein and extrinsic factors related to specific vector/treatment protocols (such as vector serotype, promoter design and expression level, and vector dose (often higher in hemophilia A to overcome challenges in FVIII expression efficiency)). Future optimization strategies must therefore focus concurrently on protein engineering (e.g., developing FVIII variants with lower immunogenicity) and advancements in vector technology (e.g., promoter optimization, capsid engineering).

## Clinical Manifestations of Hepatotoxicity

Hepatotoxicity following hemophilia gene therapy primarily presents as asymptomatic, delayed-onset transaminase elevation and is closely associated with loss of coagulation factor expression [6, 9]. Its successful management relies entirely on a proactive monitoring system centered on frequent liver biochemistry and coagulation factor activity testing, with prompt initiation of immunosuppressive therapy upon detecting abnormalities [62, 63].

### Asymptomatic transaminase elevation

The most characteristic manifestation of hepatotoxicity is an asymptomatic elevation in ALT levels. This phenomenon is highly prevalent. In the phase 1/2 clinical trial of valoctocogene roxaparvovec, the incidence of ALT elevation in the high-dose group (6 × 10^13^ vg/kg) reached 85.7% in the first year [58]; in the phase 3 trial (GENEr8-1, dose of 6 × 10^13^ vg/kg), 85.8% (115/134) of patients experienced ALT elevation and adverse events related to liver dysfunction in the first year after gene therapy infusion [25]. Its occurrence follows a distinct temporal window, primarily concentrated between 4 and 12 weeks post-infusion. Studies on AAV8-hFIX and AAV5-hFVIII-SQ both reported peak ALT elevations within this period [14, 61]. In the giroctocogene fitelparvovec (AAV6 vector) trial for hemophilia A, patients in the high-dose group also frequently developed ALT elevation within weeks post-infusion [57]. However, onset can occur as early as 2 weeks or be delayed beyond 26 weeks [25, 57]. The magnitude is mostly mild to moderate (1.5–5 × upper limit of normal (ULN)), but some patients can reach 5–10 × ULN or higher (e.g., one case in the DTX101 study had ALT of 914 U/L [64]). Importantly, the vast majority of patients are asymptomatic, and bilirubin, alkaline phosphatase (ALP), and other markers are typically normal, not meeting Hy’s law criteria.

In clinical trials of gene therapy for hemophilia, the observed hepatocellular injury is usually self-limiting, and there have been no public reports of death due to acute liver failure in this patient population. However, it must be emphasized that the risk of severe hepatotoxicity associated with AAV gene therapy is real and fatal cases have occurred [65, 66]. Although these severe events did not occur in hemophilia patients, they clearly alert to the potential risk that AAV vectors may cause life-threatening liver injury. Therefore, even in the treatment of hemophilia where liver injury usually presents as self-limiting, the highest standard of monitoring and management must be implemented to guard against severe risks.

### Decline in coagulation factor expression and the “factor loss” phenomenon

The direct functional consequence of hepatotoxicity can be the loss of transgene expression. ALT elevation often coincides with or slightly precedes a decline in coagulation factor activity, strongly suggesting a causal relationship. In AAV8-hFIX study, patients in the high-dose cohort experienced a 50–70% decline in FIX activity from peak levels during the period of ALT elevation [14]. In the AAV8 trial for hemophilia B, patients 5 and 6 exhibited expansion of AAV capsid-specific T cells concurrent with ALT peaks, accompanied by a > 50% drop in FIX levels from their peak [15]. The BAX 335 study also reported acute FIX loss due to immune responses [19].

Factor loss can be “acute,” declining from therapeutic levels to near baseline within weeks. For example, one study participant experienced a drop in FIX activity from 58.5% to baseline within 1 week of ALT elevation [19]. Alternatively, it can be “slow or gradual,” as seen in the GENEr8-1 trial where median FVIII activity decreased from 23.9 to 14.7 IU/dL over 2 years [25], potentially related to cell turnover or epigenetic silencing. In the TAK-754 (hemophilia A) study, despite a highly optimized vector genome (> 90% CpG reduction), all participants experienced ALT elevation and FVIII expression loss, indicating that this risk is pervasive [31]. Notably, when using less immunogenic serotypes (e.g., AAV5), coagulation factor levels may remain stable even in the face of ALT elevation, suggesting potential differences in the immune response mechanism or outcome [67].

## Comprehensive Monitoring Strategy

The vast majority of patients lack specific symptoms. A minority may experience non-specific fatigue or malaise, but typically without signs of severe acute liver injury like jaundice or coagulopathy [6, 9, 63]. Therefore, intervention cannot rely on symptoms and must be guided by active laboratory monitoring. Post-treatment monitoring is critical for managing safety and evaluating long-term efficacy. Recent expert consensus publications have provided salient and updated recommendations for surveillance following AAV-mediated gene therapy [8, 68, 69].

It is important to note that immune-mediated hepatotoxicity induced by gene therapy is an emerging area, and there is currently a paucity of formal, targeted clinical management guidelines issued by professional societies. Therefore, the monitoring thresholds and management strategies discussed below are primarily derived from the protocol designs of published key clinical trials, accumulated experience in managing related adverse events, and an understanding of the underlying immunological mechanisms. These recommendations represent current best practices based on limited yet important evidence and are intended to inform clinical decision-making. In practical application, healthcare teams should integrate these considerations with the patient’s specific condition, the product’s prescribing information, and the latest research evidence to make individualized judgments.

### Liver biochemistry and coagulation function monitoring

ALT and AST are the most sensitive sentinels for hepatocellular injury. Total bilirubin, gamma-glutamyl transferase (GGT), ALP, and prothrombin time (PT/INR) are used to assess the severity of liver injury and synthetic function [70, 71]. Regular measurement of FVIII:C or FIX:C is essential. Caution is needed for hemophilia B patients receiving FIX Padua variants, as their high specific activity might mask mild declines in expression.

Based on consensus from multiple clinical trials, it is recommended to monitor ALT/AST and coagulation factor activity weekly for the first 0–6 months post-AAV infusion, especially during the 0–3 month period which covers the peak incidence of immune responses. An ALT level persistently > 1.5 to 2 times the ULN is generally considered the threshold for initiating immunosuppression (e.g., oral corticosteroids). From 6 to 24 months post-infusion, the interval can be gradually extended to every 2–4 weeks. Beyond 24 months post-infusion, monitoring every 3–6 months is advised, but vigilance should be maintained due to rare reports of late immune reactions.

### Immunological monitoring

Pre-treatment screening for anti-AAV NAbs is a mandatory step for patient selection, with varying exclusion criteria across trials. Post-treatment, nearly all patients develop high-titer, cross-reactive NAbs that can persist for years to over a decade, constituting a major barrier to re-administration [38, 60]. Additionally, hemophilia A patients require periodic (e.g., monthly) monitoring for anti-FVIII inhibitors for at least 1–2 years post-infusion. Encouragingly, in large trials like GENEr8-1, only 9.0% of patients developed transient, low-titer anti-FVIII antibodies, none of which progressed to clinically significant inhibitors [61]. IFN-γ ELISpot is an important research tool for detecting AAV capsid-specific T-cell responses. In several studies, such as SPK-9001 and BBM-H901, this technique successfully detected specific T-cell activation preceding or coinciding with ALT elevation, providing a basis for predicting immune responses [45, 46]. However, due to standardization and accessibility issues, specific T-cell activation detecting is currently used more in research than routine clinical diagnosis.

### Clinical differences between hemophilia A and B

Hepatotoxicity in hemophilia B is relatively stereotypical and often responds well to corticosteroid therapy [60]. In hemophilia A, however, likely due to typically higher vector doses and potential additional metabolic stress from FVIII expression, hepatotoxicity events are more frequent and sometimes more challenging to manage, as seen in the giroctocogene fitelparvovec trial where some patients required multiple courses of corticosteroids [57]. FIX expression in hemophilia B often peaks rapidly [19, 39, 45], whereas FVIII expression in hemophilia A is typically delayed (peak at 20–28 weeks) [20, 47]. This implies that the monitoring window for immune-mediated events needs to be prolonged for hemophilia A patients, as the risk of hepatotoxicity and factor loss may extend beyond 6 months post-infusion.

### Long-term safety monitoring

Given the potential for AAV vector genome integration, long-term tumor surveillance is recommended. Most current protocols suggest annual liver ultrasound and alpha-fetoprotein (AFP) testing. It is important to emphasize that no direct link between the vector and hepatocellular carcinoma (HCC) has been identified in follow-up extending up to 15 years (e.g., the AAV2 trial) [38]. Mild hepatomegaly or fatty liver has been occasionally reported during long-term follow-up, but its causal relationship to gene therapy remains unclear [58].

## Prophylactic Strategies

To address immune-mediated hepatotoxicity and loss of transgene expression in hemophilia gene therapy, a comprehensive management strategy encompassing prevention, monitoring, and active intervention is crucial. Prevention is the first line of defense to ensure treatment success.

### Strict patient selection

Screening for pre-existing NAbs is paramount. Excluding patients with high-titer pre-existing NAbs against the specific AAV serotype used is the current standard practice globally. Although the cutoff values vary between trials and serotypes (e.g., the etranacogene dezaparvovec trial set an eligibility criterion of AAV5 NAb titer ≤ 1:678) [39], this step directly determines initial transduction efficiency and filters out individuals at the highest risk of immune responses. Furthermore, patients with active liver disease (e.g., viral hepatitis) or significant liver fibrosis (e.g., FibroScan ≥ F2) are typically excluded due to their reduced hepatic reserve and increased susceptibility to immune-mediated liver injury.

### Prophylactic immunosuppression

Routine administration of short-term (e.g., 12–24 weeks) immunosuppression post-AAV infusion to prevent or attenuate T-cell responses has become a standard practice in many centers [45, 47, 72]. Corticosteroids are the most central and evidence-backed agents. Leveraging their broad anti-inflammatory and immunosuppressive effects, oral prednisone or prednisolone is typically initiated on the day of or the day after gene infusion. The starting dose is generally 0.5–1.0 mg/kg/day. Patients are closely monitored for ALT and coagulation factor activity. If ALT remains normal and factor levels are stable, a slow taper (e.g., reducing by 5 mg every 1–2 weeks) can be initiated after 8–12 weeks, with a total course typically lasting 3–6 months. In AAV5-hFVIII-SQ studies, some patients in the high-dose cohort received prophylactic prednisone (40 mg/day, starting 3 weeks after infusion), which successfully delayed the onset and shortened the duration of ALT elevation [47]. The TAK-754 study also employed prophylactic prednisone (60 mg/day, starting on day 8) in its later stages [31]. The BBM-H901 trial (hemophilia B) pioneered initiating prednisone (1 mg/kg/day) 7 days before infusion, significantly reducing the incidence of liver enzyme elevation, indicating that pre-treatment can effectively suppress baseline immune activity [45].

### Vector engineering and optimization

Reducing immunogenicity at the technical source is a future direction. This includes selecting serotypes like AAV5, which have demonstrated lower immunogenicity and better immune evasion in clinical trials [39, 67]. Researchers are developing novel capsids or engineering existing ones to evade pre-existing immune recognition and enhance transduction efficiency, potentially allowing for lower vector doses. Furthermore, reducing the number of immunostimulatory CpG motifs in the vector genome to mitigate the risk of innate immune activation via the TLR9 pathway, a strategy emphasized in both the BAX 335 and TAK-754 studies [19, 31].

## Therapeutic Immunosuppression

Prompt intervention is mandatory upon detecting clear signs of immune activation to prevent hepatocyte damage and factor loss.

### Indications for initiating intervention

Current consensus suggests initiating immunosuppressive therapy when ALT levels are persistently > 1.5 to 2 times the ULN, regardless of the presence of clinical symptoms [6, 9, 62]. ALT elevation often slightly precedes or coincides with the decline in factor levels, making it a crucial early warning signal.

### First-line treatment: corticosteroids

High-dose oral prednisone (or prednisolone) is the established first-line regimen. The starting dose is typically 1 mg/kg/day, although fixed doses like 60 mg/day have been used in some studies [14, 15, 46]. Most patients respond favorably to corticosteroid therapy. In Nathwani’s study, ALT began to decline within 5 days of initiating treatment, with FIX levels subsequently stabilizing or partially recovering [14, 15]. In the GENEr8-1 trial, the median time to ALT decline after steroid initiation was 8 days [61]. Corticosteroids should be continued until ALT normalizes, followed by a slow tapering process, typically over 8 to 16 weeks [25, 39]. The median duration of steroid therapy reached 95 days in the BENEGENE-2 trial [59].

Although corticosteroids serve as the first-line and mainstream strategy for managing immune-mediated liver injury associated with gene therapy and can effectively suppress broad inflammatory responses, their clinical application still faces significant challenges. There is significant inter-individual variability in their efficacy, with some patients responding poorly or requiring long-term, high-dose maintenance, which leads to intolerable side effects such as hyperglycemia, osteoporosis, and endocrine/metabolic disorders. Regarding their optimal timing of initiation, dosing regimens (particularly the tapering process), and treatment duration, there remains a lack of unified consensus based on high-level evidence, often relying on clinical experience. These limitations highlight the urgent need for more precise and safer immune management strategies. Consequently, exploring alternative or adjunctive immunomodulatory approaches has become an important research direction.

### Second-line/salvage therapy

For patients with inadequate response to steroids, steroid dependence, or relapse upon tapering, second-line immunosuppressants should be added. Tacrolimus and mycophenolate mofetil (MMF) are the most commonly used options [62, 63]. Tacrolimus potently inhibits T-cell activation and IL-2 production by inhibiting calcineurin [73, 74]. MMF inhibits the proliferation of T and B cells by blocking purine synthesis required for lymphocyte proliferation [75, 76]. Tacrolimus was used in combination or as salvage therapy in the FLT180a and BBM-H901 trials [17, 45]. Intensive immunosuppression should be maintained until ALT normalizes and factor activity stabilizes, after which a slow, stepwise tapering can be attempted. Premature or overly rapid discontinuation of immunosuppression can lead to immune response “rebound.” Close monitoring for side effects of immunosuppressants, such as hyperglycemia, increased infection risk, and renal impairment, is essential throughout the treatment course.

## Managing Factor Expression Loss and Long-Term Care

### Acute phase management

If factor activity drops below the hemostatic threshold (e.g., FIX < 2%) during an immune response, short-term coagulation factor replacement therapy should be used to control bleeding. If factor expression is completely or nearly completely lost and the patient is at high bleeding risk, long-term prophylactic treatment needs to be resumed. For hemophilia A, non-factor therapies such as emicizumab represent an important alternative option [77, 78].

### Inhibitor management

Management becomes highly complex if FVIII inhibitors are detected in a hemophilia A gene therapy patient. Consideration can be given to initiating an immune tolerance induction (ITI) regimen, while using bypassing agents (e.g., recombinant factor VIIa, activated prothrombin complex concentrate) to manage bleeding events. High-dose corticosteroids combined with rituximab (an anti-CD20 monoclonal antibody for B-cell depletion) may be considered for inhibitor eradication. This requires close collaboration within a multidisciplinary team in a hemophilia treatment center.

### Long-term follow-up and safety monitoring

During the later phases of treatment and long-term follow-up, liver function and coagulation factor activity should be checked every 3–6 months to assess the long-term stability of expression [25, 39, 45]. Although there is currently no evidence directly linking AAV gene therapy to HCC [39], as confirmed by George’s 15-year follow-up and case reviews [38], annual liver ultrasound and AFP screening are still recommended based on theoretical risk [38, 58, 60]. Furthermore, patient education is essential regarding recognizing symptoms potentially related to hepatotoxicity (e.g., fatigue, jaundice) and understanding the importance of long-term follow-up.

As a single-stranded DNA virus, the genome of the AAV vector may, in rare instances, integrate into the host cell genome through mechanisms such as non-homologous end joining. Although the frequency of such events is extremely low, and the wild-type AAV itself is not oncogenic, the theoretical possibility of insertional mutagenesis provides the scientific rationale for long-term monitoring. Thus, despite accumulating evidence supporting a favorable long-term safety profile of AAV-based therapies, it remains a prevailing consensus and a regulatory expectation that patients enrolled in gene therapy trials should be entered into registries and followed for extended periods, often spanning years to decades. This practice aligns with the precautionary principle in patient safety and aims to monitor any potential delayed adverse effects.

## Current Challenges and Unresolved Issues

Despite landmark advancements, the long-term success and broad application of hemophilia gene therapy face a series of profound and interconnected challenges. These primarily stem from the complexity of immune responses, uncertainties regarding long-term safety, and inherent technical limitations.

### Unpredictability and inter-individual variability of immune responses

One of the most significant clinical challenges is the inability to accurately predict which patient will develop immune-mediated hepatotoxicity and factor loss. There is substantial interindividual variability; even among patients receiving the same vector and dose, some maintain stable expression while others experience rapid factor loss [18, 19]. This heterogeneity may be linked to individual HLA haplotype, baseline immune status, hepatic antigen presentation efficiency, and T-cell subset functionality. Concurrently, there is a lack of reliable predictive biomarkers. Pre-existing NAb titers serve only as a crude screening tool and do not perfectly correlate with clinical outcomes (e.g., AAV5 demonstrates some tolerability). The sensitivity of peripheral blood immune monitoring (e.g., ELISpot) is limited, and the local immune response within the liver is difficult to capture via circulating markers.

### Optimization and limitations of immunosuppressive strategies

Current immune management protocols are not universally effective and carry uncertainties. Prophylactic immunosuppression showed benefit in trials like BBM-H901 [45]; however, the optimal initiation timing, dosage, and total duration lack a gold standard. For reactive therapy, some patients respond inadequately to first-line corticosteroids or experience relapse upon tapering, necessitating the addition of second-line agents (e.g., tacrolimus, MMF). However, the long-term safety and efficacy of these combination regimens require further data. Moreover, immunosuppression cannot completely prevent all immune reactions and factor loss. The risks associated with long-term or high-dose corticosteroid use, including metabolic disturbances, osteoporosis, and infections, warrant particular attention in the hemophilia population.

### Uncertainties in long-term safety

The lifelong safety profile of gene therapy requires decades of follow-up for confirmation. Although AAV vectors predominantly persist as episomes, the theoretical risk of low-frequency integration and potential tumorigenesis remains. While the longest follow-up to date (15 years for AAV2) and some clinical studies have not identified vector-related HCC [38, 39], the lifetime risk associated with higher vector doses (e.g., ≥ 6 × 10^13^ vg/kg) remains unknown, necessitating long-term data accumulation through international registries (e.g., WFH Gene Therapy Registry) [79]. Furthermore, it is unclear whether sustained, low-level immune activity or hepatocyte stress could lead to occult liver fibrosis, mandating lifelong monitoring.

### Challenges in special populations

Current gene therapy research focuses predominantly on adult hemophilia patients. The more active immune system and ongoing liver growth in children could lead to more robust immune responses or accelerated vector DNA loss due to hepatocyte division; defining the efficacy-safety balance in this population is a critical future research focus. Furthermore, patients with chronic liver conditions such as HCV infection or fatty liver disease, who are at potentially higher risk for hepatotoxicity, are typically excluded from current therapies, limiting treatment accessibility.

### The formidable barrier of pre-existing immunity and re-administration

This is widely considered one of the most severe challenges in the field. Approximately 30–50% of adults are excluded from therapy due to pre-existing AAV NAbs, significantly limiting the broad applicability of gene therapy. Furthermore, the high-titer, cross-reactive NAbs generated post-therapy can persist for years to over a decade [38], effectively creating a permanent barrier to re-administration using the same or similar serotypes. For patients with suboptimal initial efficacy or those whose factor expression wanes over time due to natural cell turnover, there are currently no effective re-treatment options.

## Conclusions

Hemophilia gene therapy represents a revolutionary therapeutic breakthrough, offering patients the potential for liberation from lifelong replacement therapy. However, the long-term efficacy and safety of AAV-mediated, liver-directed gene therapy remain constrained by host immune responses. Immuno-mediated hepatotoxicity and the consequent loss of coagulation factor expression constitute the primary biological barriers currently faced by this treatment modality. This process is initiated by the activation of the innate immune system (e.g., via the TLR9/cGAS-STING pathway) following the infusion of high-dose AAV vectors. This subsequently triggers an adaptive immune response, predominantly driven by AAV capsid-specific CD8^+^ T cells, through cross-presentation by antigen-presenting cells. The recognition and elimination of transduced hepatocytes by these cytotoxic T lymphocytes directly lead to asymptomatic transaminase (ALT) elevation and a progressive decline in coagulation factor levels.

Centered on this core mechanism, a relatively clear clinical management pathway has been established. Its cornerstone involves stringent patient selection prior to therapy (excluding individuals with high-titer pre-existing neutralizing antibodies and severe liver disease), the initiation of corticosteroid-based prophylactic or therapeutic immunosuppression, and a long-term follow-up system primarily based on frequent monitoring of ALT and coagulation factor activity. Upon detection of immune activation, promptly escalating the immunosuppressive regimen is crucial for rescuing factor levels.

Despite continuous refinements in management strategies, numerous challenges persist. These include the difficulty in predicting individual variations in immune responses, the hurdles posed by pre-existing immunity and re-administration, uncertainties regarding long-term safety, and differences in expression durability and immune risks between hemophilia A and B. Addressing these challenges relies on synergistic innovation across multiple fields. This includes developing next-generation vectors with lower immunogenicity and non-viral delivery platforms to circumvent risks at the source; implementing biomarker-guided precision immune management for individualized treatment; and exploring diverse platforms such as gene editing and non-liver-targeted strategies to expand the therapeutic landscape.

In conclusion, a profound understanding and effective management of immune-mediated hepatotoxicity are paramount to ensuring the success of hemophilia gene therapy. With ongoing advancements in gene therapy research, we are poised to overcome these obstacles, offering safer, more effective, and durable treatment options for people with hemophilia worldwide.

## Data Availability

All data generated or analyzed during this study are included in this published article.

## Abbreviations

AAV: adeno-associated virus
AFP: alpha-fetoprotein
ALP: alkaline phosphatase
ALT: alanine aminotransferase
GGT: gamma-glutamyl transferase
HCC: hepatocellular carcinoma
IFN-γ: interferon-γ
ITI: immune tolerance induction
MMF: mycophenolate mofetil
NAbs: neutralizing antibodies
PAMPs: pathogen-associated molecular patterns
PT: prothrombin time
TLR9: Toll-like receptor 9
